# A Pilot Randomized-Controlled Trial on the Effect of CPAP Treatment on Glycemic Control in Gestational Diabetes: Study Design and Methods

**DOI:** 10.3389/fendo.2018.00659

**Published:** 2018-11-16

**Authors:** Sushmita Pamidi, Sara J. Meltzer, Natasha Garfield, Lorraine Lavigne, Allen Olha, Ahamed Khalyfa, Andrea Benedetti, Geneviève Tremblay, Robert Gagnon, Evelyne Rey, Kaberi Dasgupta, R. John Kimoff

**Affiliations:** ^1^Division of Respiratory Medicine, Department of Medicine, McGill University Health Centre, McGill University, Montreal, QC, Canada; ^2^Division of Endocrinology, Department of Medicine, McGill University Health Center, Montreal, QC, Canada; ^3^Department of Epidemiology, Biostatistics & Occupational Health, McGill University, Montreal, QC, Canada; ^4^Department of Obstetrics and Gynecology, McGill University, Montreal, QC, Canada; ^5^Department of Medicine, CHU Sainte-Justine Research Center, Université de Montréal, Montreal, QC, Canada

**Keywords:** sleep apnea, diabetes, CPAP (continuous positive airway pressure), gestational diabetes, pregnancy

## Abstract

**Background:** Gestational diabetes (GDM) is associated with adverse short- and long-term maternal and fetal outcomes. Observational data support a link between sleep-disordered breathing (SDB) during pregnancy and GDM. However, it is unknown whether treatment of SDB with continuous positive airway pressure (CPAP) improves glucose control in this patient population. In addition, CPAP adherence and feasibility as a treatment option in pregnancy is unknown. This pilot randomized, controlled trial aims to primarily determine the feasibility of CPAP treatment in pregnant women with SDB and GDM. This study is also investigating the effect of SDB treatment on 24-h glucose profiles as an exploratory outcome.

**Objectives:** To describe the study methodology in this ongoing study of pregnant women with GDM and SDB.

**Patients and Methods:** Pregnant women with GDM and SDB defined by apnea-hypopnea index (AHI) ≥10 (Chicago Scoring Criteria) on level 2 polysomnography are randomized to either auto titrating CPAP (experimental group) or a nasal dilator strip (control group) until delivery. The primary outcome, objectively-assessed adherence to CPAP, is measured over the course of the treatment period using device-specific software. Recruitment and retention rates will be calculated to assess the feasibility for planning future trials. Twenty-four hour glucose profiles are measured over a 72-h period using the continuous glucose monitoring (CGM) system, before and after the intervention.

**Conclusion:** The results of this study will be highly informative to determine whether CPAP is a feasible treatment for pregnant women with GDM and SDB, a specialized population at risk for substantial comorbidity. The trial results will ultimately be useful in planning future SDB treatment trials in pregnancy and GDM.

The study is registered on clinicaltrials.gov (NCT02245659).

## Introduction

Gestational diabetes (GDM) is glucose intolerance that is first recognized during pregnancy (1). The prevalence of GDM doubled between 1994 and 2002 in a multi-ethnic U.S. population (2), possibly related to the global epidemic of obesity. Current estimates of prevalence range from 8 to 16%, depending on the study population and diagnostic criteria used (3, 4). Maternal hyperglycemia can lead to both short-term and long-term adverse outcomes for both the mother and baby, including increased rates of preeclampsia, perinatal mortality, cesarean section, neonatal metabolic abnormalities, macrosomia and resulting birth injuries (5). In addition, in the long-term, a history of GDM in women is associated with an increased risk of developing cardiovascular disease (6–8), type 2 diabetes (9, 10) and non-alcoholic fatty liver disease (NAFLD) (11, 12). Although the majority of GDM cases arise during pregnancy and resolve after delivery, long-term follow-up studies demonstrate that ~20–50% of women progress to type 2 diabetes, a risk which has doubled over the past 10 years (13–16). Moreover, there is also an increased risk for developing metabolic syndrome and being overweight later in life among offspring exposed to GDM (17–20). Thus, prevention and improved management of GDM may improve outcomes for both the mother and offspring.

Sleep-disordered breathing (SDB) is prevalent in 17–45% of pregnant women by the third trimester (21–25), depending on the diagnostic cut-offs used, degree of obesity, and comorbidities present in the study population. SDB is characterized by breathing pauses during sleep, which results in sleep disruption from frequent arousals and intermittent hypoxia. Similar to polysomnography-based estimates of SDB prevalence, symptoms of SDB are reported in 14–35% of pregnant women by the 3rd trimester (26–28). In addition to weight gain, pregnancy-specific physiological changes that include upper airway narrowing, vascular congestion, nasal congestion and decreased functional residual capacity (29, 30) are hypothesized to increase the risk of SDB. In several studies that have adjusted for obesity, SDB in the non-pregnant population is associated with glucose intolerance and type 2 diabetes (31, 32). Although the specific mechanisms of this relationship have yet to be clearly defined, pathways involving increases in sympathetic drive, cortisol, and inflammation, are likely involved (33, 34). Extrapolating from the non-pregnant literature, SDB may also represent a novel risk factor for GDM (21, 31, 35). Two recent meta-analyses of observational studies demonstrated an increased risk of GDM in maternal SDB (36, 37) (adjusted odds ratios of 1.86–3.06). More recently, publication of the nuMoM2b cohort sleep substudy, which assessed SDB in pregnancy in the largest cohort to date (*n* = 3,705), revealed similar results between the presence of SDB in pregnancy and the risk of GDM (odds ratios ~3) (38).

Despite the accumulating evidence that gestational SDB is associated with GDM, the data are limited by the biases inherent in observational studies, particularly with possible residual confounding from obesity. Performing controlled interventional studies with treatment of SDB is the next essential step to determine whether there is an associated improvement in outcomes. Our ultimate objective is to perform a large-scale, multi-center randomized trial evaulating the effects of CPAP treatment of SDB on glycemic control in GDM. To date, a few studies, mostly uncontrolled and all with small sample sizes, have investigated whether treatment of SDB with CPAP improves blood pressure among patients with gestational hypertension or preeclampsia [Reviewed in (21)]. In a recently published trial of CPAP vs. no CPAP for 2 weeks in women with GDM, of the 15 women on CPAP, only 7 were adherent (minimum usage 4h/night for > 70% of nights) (39). However, adherence to CPAP for a longer duration during pregnancy and its effect on 24-h glucose profiles is unknown.

Several CPAP trials in non-pregnancy that have failed to demonstrate improvements with respect to metabolic outcomes have also been limited by poor CPAP adherence [reviewed in (31)]. Optimizing CPAP use, on the other hand, has improved cardiometabolic outcomes (40–45), particularly in in-laboratory proof-of-concept studies with 8 h a night of CPAP use in patients with prediabetes (46) and type 2 diabetes (47). In pregnancy, CPAP adherence may be further affected by the unique sleep complaints that are prevalent in pregnancy, including less deep sleep and more frequent nocturnal awakenings (48). In order to appropriately power large, multi-center trials in pregnant women with SDB for improving cardiometabolic outcomes, the adherence to CPAP among pregnant women needs to first be established. As such, the primary aim of this pilot trial is to assess the adherence to CPAP in pregnant women with GDM, a unique population at risk for significant comorbidity. The secondary aims are to collect pilot data on glycemic control in the form of 24-h continuous glucose monitoring (CGM) as well as other relevant outcomes to allow planning and sample size calculations for a future, multi-center trial with the primary aim of evaluating the effects of SDB treatment on glycemic control in GDM.

## Methods

### Trial protocol

**Figure 1:**
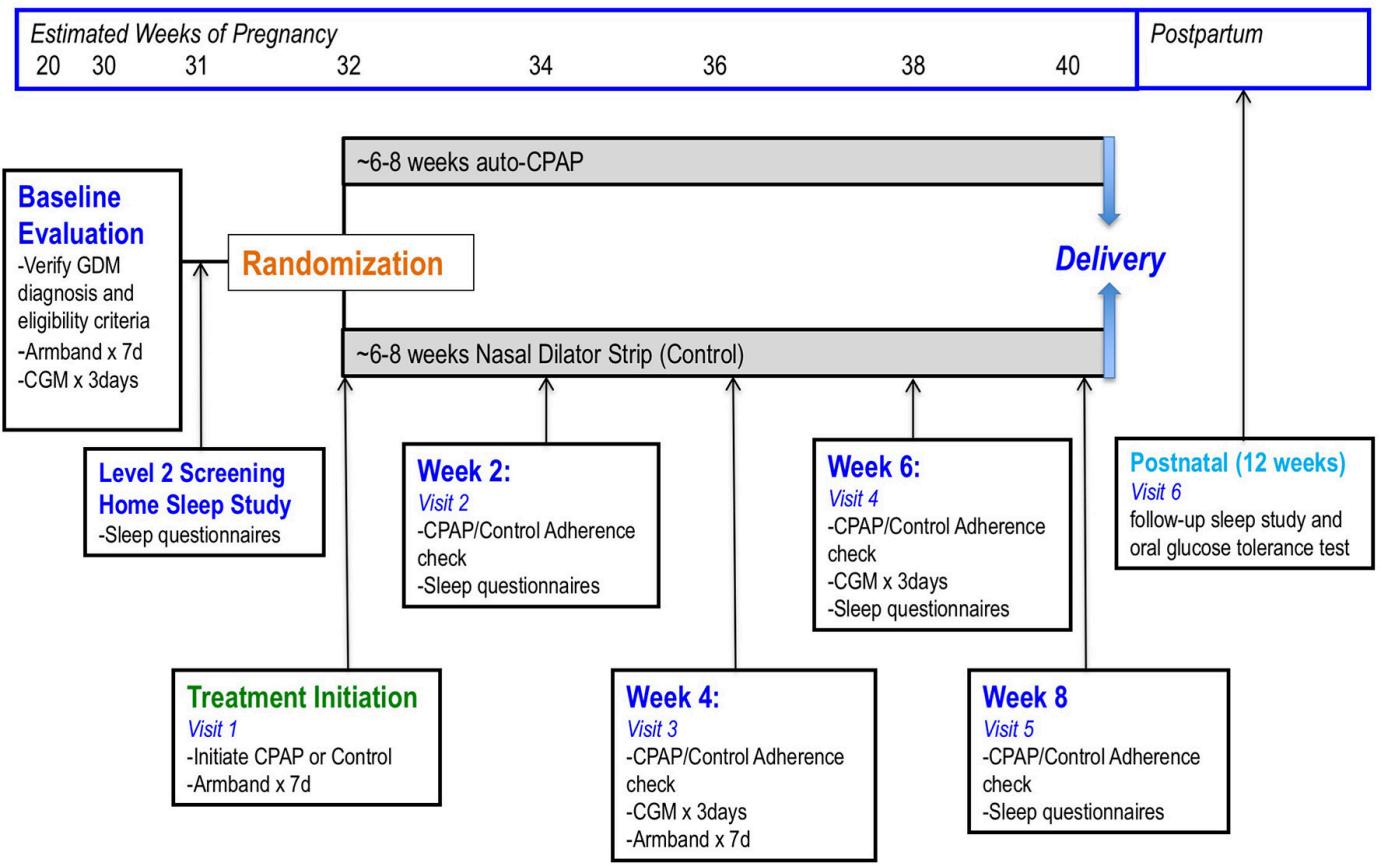
The trial protocol involves a baseline evaluation that initially confirms the diagnosis of gestational diabetes (GDM). This testing usually takes place between 24 and 28 weeks of gestational age. During the baseline visit, participants wear an Armband, consisting of an accelerometer, for 1 week in order monitor activity levels and sleep-wake patterns. Insertion of a continuous glucose monitoring (CGM) device for 72 h takes place at this visit. Within 1–2 weeks, a home sleep study to screen for sleep-disordered breathing (SDB) is organized. If participants are eligible for the study, randomization to either auto-titrating continuous positive airway pressure (CPAP) or nasal dilator strip (control group) takes place. Participants follow-up with the study nurse every 2 weeks to verify adherence to therapy and complete study measurements. Post-partum, participants are invited back for a repeat sleep study ~12 weeks after the study is completed and also complete an oral glucose tolerance test as part of routine follow-up with the endocrinologist.

This is an unblinded, randomized-controlled parallel study. Figure 1 demonstrates the protocol design and testing at each visit. Recruitment started in March 2015 at two centers in Montreal, Canada (McGill University Health Centre and Centre Hospitalier Universitaire Sainte-Justine).

### Participants

Participants are screened for eligibility if they are pregnant women aged ≥18 years referred to the GDM clinic between >20 and <34 weeks gestational age with a diagnosis of GDM. Participants receive a diagnosis of GDM prior to their first study visit based on a positive non-fasting screening 50-g glucose load (24–28 weeks gestation) of >11.1 mmol/L. If this test is abnormal but not in the diabetic range (7.8–11.1 mmol/L), then a fasting, standard, 75-g oral glucose tolerance test (OGTT) is ordered. GDM is defined at our center with either an abnormal 50-g screening test with level ≥11.1 mmol/L or from one of the following from the 75-g OGTT: (1) fasting glucose ≥5.1 mmol/L, (2) 1-h glucose ≥10.0 mmol/L, or (3) 2-hr glucose ≥8.5 mmol/L (49), all in the absence of pre-existing or pregestational diabetes. Screening questions on snoring, witnessed apneas or other symptoms of SDB are not used in the selection process. Participants are not eligible if they had multiple pregnancy, current cigarette smoking or alcohol consumption, chronic renal disease, cardiovascular disease, stroke, active psychiatric disease, active malignancy, HIV infection, Hepatitis C or B, prior treatment for SDB, occupation involving shift work or travel across time zones, or inability to provide informed consent.

Within 1 week of the initial GDM clinic visit, participants are scheduled for a one-night level 2 screening home polysomnogram. A sleep technologist sets up the complete polysomnogram (Titanium unit, Medcare, Natus Inc., Mississauga, ON) in the participant's home in the evening and verifies all signals. Following the sleep recording, a driver picks up the device from the participant's home and returns it to our sleep laboratory. Data from the recorder is downloaded and studies are scored by a Registered Polysomnographic Technologist with review by one of the study sleep physicians (SP, RJK). Standard quality assurance measures for scoring reliability are applied including ensuring a minimum of ~4 h of total sleep time. In addition, oximetry, electroencephalography (EEG), and nasal cannula signals are verified for adequate quality, which is necessary for accurate scoring of respiratory events. Sleep-wake state, arousals and periodic limb movements are scored in accordance with current AASM criteria (50); respiratory events are scored using standard Chicago criteria (51). A diagnosis of SDB establishing eligibility for randomization is made based on the presence of an apnea-hypoponea index (AHI) ≥10 based on Chicago respiratory scoring criteria. Since pregnancy is generally characterized by milder SDB and lesser degrees of oxygen desaturation (21, 52–55), the more sensitive Chicago scoring criteria were chosen for diagnosis of SDB.

Potential participants in whom sleep studies demonstrate severe SDB (AHI ≥30 events/h) with significant sleepiness (ESS ≥15), or significant oxygen desaturation regardless of symptoms (4% oxygen desaturation index ≥30 or sustained hypoxia <80%) are excluded and sent for urgent evaluation at the Sleep Clinic at our institution.

### Randomization

Eligible participants are randomized using web-based randomization with permuted blocks of varying size (Dacima software, Montreal, Quebec).

### Treatment interventions

#### Experimental group

Nightly CPAP treatment until delivery: In the participants randomized to the active intervention arm, auto-titrated CPAP (auto-CPAP) is started within 2 weeks of the initial GDM clinic visit following a positive sleep study for SDB. Active arm participants receive an individualized 1 h session on set-up, mask-fitting and preference, and potential side-effects (e.g., dry mouth, nasal congestion). A variety of nasal masks are initially tried for comfort during set-up, but if mouth-breathing, or intolerance to the nasal mask occurs, an oronasal mask is fitted. Education, troubleshooting of side effects occurs every 2 weeks, and more often as necessary by telephone or in-person visits. Download of the CPAP adherence data (adherence, leaks, pressure settings, efficacy with residual AHI) occurs either by Wi-Fi using available software or by interrogation of the memory chip at specific visits every 2 weeks (Figure 1) (56). All participants assigned to CPAP are encouraged to use the device for the remaining period of pregnancy (~8 weeks) for as many hours during sleep as possible.

#### Control group

Significant uncertainty exists on the optimal control group for CPAP studies. In particular, in pregnant women, a sham CPAP group may likely interfere with sleep quality and may shorten sleep duration. These effects may worsen glucose control and bias the results in favor of the active intervention arm. For these reasons, nightly nasal dilator strips are being tested in this population as a possible control. The strips (Breathe Right®, GlaxoSmithKline, Brentford, UK) mechanically pull the lateral nasal walls outward, causing nasal passage dilatation, thereby easing breathing and reducing snoring, but not SDB, in the general population (57). Since nasal strips have not been evaluated thoroughly in pregnancy, we are assessing for adherence in a way that is analogous to a pill count, by monitoring for leftover strips. Side effects and tolerability is also queried during each clinic visit. Control arm participants also complete a level 2 home polysomnogram 2 weeks after starting the nasal strip to determine if any improvements in respiratory parameters occurred.

### Follow-up visits

#### Antenatal

The GDM clinics at the recruitment sites routinely follow patients with GDM every 2–4 weeks from diagnosis until delivery. During these clinic visits, participants receive their routine care from their endocrinologist, nurse, maternal-fetal-medicine specialist and dietitian. Participants are then seen by our research team during the same visit if possible to verify and record CPAP adherence, download CGM and capillary blood glucose measurements when appropriate, and assess sleep habits through questionnaires. Figure 1 indicates the specific tests and evaluations that are performed at each scheduled visit.

#### Postnatal

After delivery, neonatal and obstetrical outcomes are abstracted through chart review. All study participants are referred to the Sleep Clinic between 1 and 3 months post-partum for assessment of persistent SDB symptoms and a repeat sleep study. Participants also have a follow-up in the GDM clinic with an OGTT to assess for post-partum diabetes and prediabetes. Treatment with CPAP is offered to participants in both groups after delivery until SDB is reassessed at the postnatal Sleep Clinic visit.

### Outcomes

1. The primary outcome in this study is to objectively determine the adherence to CPAP among women with GDM and SDB. Acceptable adherence is defined by mean usage ≥4 h/night for at least 70% of nights, the conventional threshold for acceptable CPAP adherence in the general non-pregnant population (40–42, 58). Secondary analyses will examine predictors of CPAP adherence (i.e., demographic and SDB severity variables).
2. As a secondary outcome, the suitability of nasal dilator strips as a possible control intervention for a future large-scale, multi-centered RCT in GDM will be assessed. Patient adherence, changes in indices of SDB as assessed by level 2 polysomnography, and changes in sleep quality and daytime sleepiness (questionnaire-based assessments) will be measured.
3. Recruitment and retention rates will be computed at the end of the trial. This will be important in planning a future large scale RCT.
4. The feasibility of measuring glucose levels using CGM in CPAP vs. control groups is also being assessed. CGM provides in-depth information on glucose levels that cannot be obtained from routine blood glucose measurements. We use the well-validated iPro2® (Medtronic, Northridge, CA) CGM (59, 60), a small, minimally invasive device with a painless, subcutaneous sensor measuring interstitial glucose levels every 5 min. These levels are comparable to venous blood glucose measurements (61–63). A trained technician inserts the CGM device subcutaneously in the arm or abdomen, which takes measurements over 3 days to take into consideration day-to-day variability (64). CGM has been used in pregnant subjects previously and is well-tolerated (65). The CGM is blinded so participants cannot see glucose values and will be not be burdened by alarms or sensor inaccuracy. To ensure accuracy of the measurements, CGM requires calibration against capillary blood glucose measurements that are measured as part of standard of care 4 times/day by the participant (61). Insulin requirements are documented at each visit using self-reported insulin doses in patient logbooks. Participants are asked to maintain their usual diet and exercise patterns during this time. Glucose levels between the treatment and control groups will be compared.

### Other assessments

#### Sleep quality and duration

Short sleep duration and sleep quality are also assessed, as these can be additional factors contributing to glucose dysregulation (66, 67). The Pittsburgh Sleep Quality Questionnaire (PSQI) (68) Berlin Questionnaire (69) and Epworth Sleepiness Scale (70) are administered at baseline, and again at weeks 2 and 6 after treatment initiation. Sleep time is objectively measured with the Sensewear® Armband (BodyMedia), which uses an actigraphy analysis to record movements for estimating sleep-wake activity. This Armband is worn for 1 week both at baseline and 1 month after treatment initiation. The pilot study is intended to assess the feasibility of performing these measurements in the pregnant GDM population with SDB.

#### Activity and diet assessments

Dietary habits, including self-reported total energy intake and macronutrients consumed (e.g., fats, protein, carbohydrates) are assessed through routine clinically-administered dietary logs that are monitored by the clinic dietician every 2 weeks. Body weight is recorded at each visit. Moreover, we also measure levels of physical activity through the Sensewear® Armband analysis, which is validated for and measures levels of physical activity (steps, average mets, and energy expenditure) (71, 72). The Armband is worn for 1 week at baseline and after treatment initiation as indicated in Figure 1.

#### Maternal-fetal outcome data

The medical records for each participant is reviewed to obtain data regarding the perinatal course, including details on labor and delivery, post-partum complications, and the course and complications in the newborn.

### Sample size

The assumption underlying the calculation of the sample size was that for CPAP to be an acceptable treatment option in pregnant women with GDM, the adherence should be similar to that observed in the non-pregnant population. Interventional studies with CPAP in non-pregnant populations have demonstrated a wide range of adherence rates [46–83% (73, 74)], with a few recent trials demonstrating adherence rates of ≥60–70% (40, 41). We estimate that 30 participants (CPAP group) will be needed to observe a CPAP adherence rate of 70% with a confidence interval of width of 0.34 or less with probability > 90%. In Table 1, the confidence interval width for CPAP adherence is estimated based on various sample sizes that may be achieved with the pilot trial, with a probability >90%.

**Table 1:** Confidence interval widths for achieving CPAP adherence rates of 70% based on sample size per group achieved in pilot trial.

**Sample size**	**Confidence interval width, with probability >0.90**
20	0.36
25	0.36
30	0.34

### Data analysis

Between-group differences will be assessed for statistical significance with the Student's *t*-test for normally distributed variables and the non-parametric Mann-Whitney test for non-normal distributions. Continuous variables will be reported as mean ± standard deviation. Median and interquartile ranges will be used for non-normally distributed variables. The primary outcome assesses CPAP adherence, which will be reported as % acceptable adherence rates with associated 95% confidence intervals (using conventional definition of ≥4 h/night for >70% of nights) in participants randomized to the CPAP intervention group. Adherence in the control group will be calculated as the proportion of nights the Breathe Right strips are used (total number of strips used from box over period of treatment nights). Secondary analyses will include the predictors of CPAP acceptable adherence in this population (e.g., demographic and SDB severity variables, SDB sleep symptoms). Recruitment rates will be calculated as the number of participants randomized at each site over the enrolment period. Retention rates will be 100 minus the percent of participants that dropped out after randomization over the period of the trial. Missing data will be analyzed by multiple imputation.

For examination of between-group differences in glycemic control, an intention-to-treat analysis is planned. At baseline and follow-up assessments, mean (standard deviation) 24 h, daytime, and nighttime glucose values will be computed as well as time that glucose levels are above 7.8 mmol/L (hyperglycemia). Between-group differences in change in glycemic control will be calculated with 95% CI. Secondary analyses will estimate linear regressions and adjust for relevant covariates (age, sex, BMI, baseline AHI, baseline glucose).

To minimize bias in interpreting results, the statistician and researchers involved in outcome assessment during data collection and analyses will be blinded to treatment arm allocation.

## Discussion

In light of observational data demonstrating robust associations between SDB and the risk of GDM and gestational hypertension (22, 36–38, 75) interventional controlled trials are necessary to assess the direct impact of SDB treatment on improving outcomes related to GDM. Several cardiometabolic trials in non-pregnancy with equivocal or negative results have had barriers in adequate CPAP adherence (76, 77), while other studies ensuring full CPAP compliance have demonstrated improvement in glucose levels using overnight or 24-h glucose monitoring (46, 47, 78). This pilot trial in SDB and GDM therefore focuses on CPAP adherence as the primary outcome.

Although the glycated hemoglobin (HbA1c) is the conventional measure of glucose in clinical practice in patients with type 2 diabetes, this measure does not capture changes that may occur specific to time of day (i.e., nocturnal vs. daytime), post-prandial measurements, general levels of glycemic variability throughout the day, and time spent in hyperglycemia. Moreover, since HbA1c reflects glycemia over several weeks, potentially important changes in glucose levels over a shorter period of time, as may occur during pregnancy, may be missed. CGM has been recently used in clinical trials in patients with T2DM to more specifically characterize the effect of newer therapies on various aspects of glucose control (79, 80). Of note, maintaining normal glucose levels with 24-h monitoring in GDM has been shown to result in improved maternal and fetal outcomes (81).

The results of this trial will provide novel data on the feasibility and acceptance of CPAP treatment of SDB among women with GDM and will provide valuable preliminary findings on which aspects of glucose control, if any, are improved by CPAP in pregnancy. These data will be essential to planning future, larger RCTs to definitively evaluate the impact of SDB treatment during pregnancy on maternal and infant outcomes.

## Ethics and consent

All participants provide written, informed consent in order to undergo the screening sleep study and protocol. The study is approved by the Research Ethics Board of the McGill University Health Centre and Centre Hospitalier Universitaire Sainte-Justine. All adverse events are reported by the study PI (SP) to the Research Ethics Board using standard procedures. Appropriate steps are immediately taken to deal with adverse events including sending affected participants for medical evaluation as indicated.

## Author contributions

SP conceived of the study design, managed and led the study, wrote the manuscript and was involved in all editing. SM and KD helped with study design and editing of manuscript. NG and RG helped with study design and participant recruitment. LL, AO, AK, and GT helped with study conduct. AB helped with study design and statistical analyzes. ER helped with study design and participant recruitment. KD helped with study design, and editing. RK helped with study design, study conduct, and editing.

### Conflict of interest statement

The authors declare that the research was conducted in the absence of any commercial or financial relationships that could be construed as a potential conflict of interest. The reviewer JT-H and handling Editor declared their shared affiliation. The CPAP devices used for this research study were obtained at a reduced cost from Vitalaire.
